# Effects of Raloxifene on Bone Metabolism in Hemodialysis Patients With Type 2 Diabetes

**DOI:** 10.5812/ijem.3794

**Published:** 2012-04-20

**Authors:** Osamu Saito, Takako Saito, Shinji Asakura, Tetsu Akimoto, Makoto Inoue, Yasuhiro Ando, Shigeaki Muto, Eiji Kusano

**Affiliations:** 1 Department of Nephrology, Jichi Medical University, Tochigi, Japan; 2 Oyama Suginoki Clinic, Tochigi, Japan

**Keywords:** Diabetes Mellitus, Type 2, Hemodialysis, Osteoporosis, Raloxifene

## Abstract

**Background:**

Osteoporosis and chronic kidney disease are common conditions in older adults, and often occur concurrently. Bone disease is caused by increased bone turnover accompanying secondary hyperparathyroidism, and by factors such as bone metabolic disorder accompanying kidney disease and postmenopausal or age-related osteoporosis, even in hemodialysis patients. Raloxifene is commonly used for the treatment of postmenopausal osteoporosis in the general population, and may be a treatment option for osteoporosis in hemodialysis patients. However, the effects of raloxifene in hemodialysis patients with type 2 diabetes have not been examined in detail.

**Objectives:**

This study was performed to investigate the effects of raloxifene on bone turnover markers and bone density in postmenopausal women with type 2 diabetes mellitus who were undergoing hemodialysis in Japan.

**Patients and Methods:**

The subjects were 60 female patients on maintenance hemodialysis (non-diabetic, n=30; diabetic, n=30). Raloxifene hydrochloride (60 mg) was administered to 14 diabetic patients and 14 non-diabetic patients for one year, and these patients were compared with control groups (no raloxifene) of 16 diabetic patients and 16 non-diabetic patients. Serum levels of N-terminal cross-linking telopeptide of type I collagen (NTx), bone alkaline phosphatase, and intact parathyroid hormone (iPTH) were measured, and bone density was determined by quantitative heel ultrasound at the speed of sound (SOS) in the calcaneus during this period.

**Results:**

There were no significant differences in the levels of bone turnover markers except for iPTH after treatment of diabetic and non-diabetic patients with raloxifene for one year. SOS increased after treatment with raloxifene, but was significantly decreased in the control groups. Treatment with raloxifene resulted in a significant decrease in NTx and a significant increase in SOS in both diabetic and non-diabetic patients. There were no significant differences between the diabetic and non-diabetic patients who received raloxifene.

**Conclusions:**

Treatment with raloxifene can suppress reduction in bone density in postmenopausal women with type 2 diabetes who are undergoing hemodialysis.

## 1. Background

Osteoporosis and chronic kidney disease (CKD) are common conditions in older adults, and both may be associated with hypertension and diabetes. Older persons are likely to have some degree of CKD and low bone mineral density (BMD). Hemodialysis patients (CKD stage 5D) typically have renal osteodystrophy, reflecting increased bone turnover accompanying secondary hyperparathyroidism. However, bone disease in hemodialysis patients is caused by factors such as bone metabolic disorder accompanying kidney disease and postmenopausal or age-related osteoporosis, as well as by renal osteodystrophy. Adynamic bone disease is common in hemodialysis patients with diabetic mellitus (1), and diabetes mellitus also has a major influence on bone metabolism in hemodialysis patients.

The metabolic and endocrine changes in diabetes may adversely affect bone quantity and/or quality, and increase the risk of fractures in the general population. Diabetic patients with normal kidney function typically have low bone turnover with reduced bone formation and, to a lesser degree, bone resorption (2). A decrease in bone formation markers such as serum osteocalcin, and an increase in bone resorption markers such as serum tartrate-resistant acid phosphatase occurs in type 1 diabetes mellitus (3). Patients with type 2 diabetes are at high risk of bone fracture even if their BMD is normal or high, and this increased risk of fractures may be explained by poor bone quality (4). Sharifi et al. found that a higher level of HbA1c, a marker of blood glucose control, was related to lower lumbar spine density in diabetic women (5).

Amenorrhea and early menopause often occur in female hemodialysis patients due to hypoestrogenemia, and Weisinger et al. found that bone volume is decreased in these patients (6). These results suggest the involvement of estrogen deficiency in bone disease in female hemodialysis patients. Raloxifene hydrochloride is a selective estrogen receptor modulator (SERM) that has a lower carcinogenic risk than estrogen and beneficial effects on bone (7). Raloxifene is commonly used for the treatment of postmenopausal osteoporosis in the general population, and the use of raloxifene for hemodialysis patients has also been reported (8, 9). We previously showed that raloxifene improves low BMD in patients around the time of menopause, as well as in postmenopausal hemodialysis patients of advanced age (10). Thus, this drug may also be a treatment option for osteoporosis in hemodialysis patients, but the effects of raloxifene in hemodialysis patients with type 2 diabetes have not been examined in detail.

## 2. Objectives

The goal of this study was to compare changes in bone turnover markers and bone density over one year in female hemodialysis patients with type 2 diabetes who did and did not receive treatment with raloxifene. The changes in these parameters were also compared in similar groups of female hemodialysis patients who did not have type 2 diabetes.v

## 3. Patients and Methods

### 3.1. Study Design

The subjects were 65 patients who underwent maintenance hemodialysis at Oyama Suginoki Clinic, Japan, between April 2007 and December 2009. The study was conducted according to the principles of the 1975 Declaration of Helsinki. We obtained permission for the study from the local ethical committee of our institution, and all the patients provided their written informed consent prior to participation. Of the 65 patients, 5 were excluded because they had changes in their intact parathyroid hormone (iPTH) of more than ± 50% in the 4-month observation period, and we wished to avoid the influence of a large change in iPTH. For the remaining 60 patients, age, menstrual history, duration of dialysis, and causes of renal failure were recorded. All were postmenopausal females with a mean (± SD) age of 66.1 ± 10.9 years (range: 50–87 years). The patients had been undergoing hemodialysis for 4 months to 20.2 years (mean duration: 5.1 ± 5.0 years), and were being treated twice or thrice weekly with standard bicarbonate dialysis using semisynthetic membranes (dialysis filter surface area: 1.3–2.0 m^2^). Dry weight was targeted in each case to achieve a normotensive edema-free state. Thirty patients had diabetic nephropathy and 30 had non-diabetic nephropathy. All diabetic patients had type 2 diabetes, with 3 receiving insulin therapy, 4 taking sulfonylurea drugs, and 4 taking an α-glucosidase inhibitor. The remaining 19 diabetic patients were on diet therapy alone (i.e., were without drug therapy). The diabetic and non-diabetic groups of patients were randomly divided into a raloxifene group (n=14 each), in which 60 mg of raloxifene was administered daily after the evening meal, and a control group (n=16 each), in which patients did not receive raloxifene.

In all four groups, pre-dialysis serum levels of calcium (Ca), phosphorus (P), iPTH, N-terminal cross-linking telopeptide of type I collagen (NTx; a bone resorption marker), and bone alkaline phosphatase (BAP; a bone formation marker), were measured prior to treatment and at one year after treatment initiation. Serum iPTH was measured by IRMA (Allegro Intact PTH; Nichols Institute) with an intraassay CV of 4.8%. Serum NTx was measured by ELISA (Osteomark NTX serum; Ostex International) with an intraassay CV of 4.6%. Serum BAP was measured with an enzyme immunoassay (ALK-PHASE-B; Metra Biosystems) with an intraassay CV of 2.2% (11). Other parameters were measured using standard laboratory methods.

Bone density was evaluated by quantitative heel ultrasound measured at the same time with a CM-100 ultrasound densitometer (Furuno Electric Co., Hyogo, Japan) after each hemodialysis session. The CM-100 measures the speed of sound (SOS) in the calcaneus with an intraassay CV of about 0.2%. The SOS value has been shown to be significantly and positively correlated with lumbar BMD in the general population using methods such as dual-energy X-ray absorptiometry (DEXA) (12). The diagnostic criterion of osteoporosis is a SOS value < 1479 m/s and the diagnostic criterion of osteopenia is a SOS value < 1501 m/s (12). In dialysis patients, SOS obtained from quantitative heel ultrasound has a significant correlation with BMD obtained by DEXA, although the sensitivity and specificity of the SOS value is slightly lower than that in healthy subjects (13).

### 3.2 Statistical Analysis

Results are shown as means ± standard deviation. Differences between diabetic and non-diabetic patients were analyzed by Mann-Whitney U-test (continuous variables) or Chi-squared analysis (categorical variables). Differences between the control and raloxifene groups were also analyzed by Mann-Whitney U-test (continous variables). All analyses were performed using StatView ver. 5.0® (Abacus Concepts, Inc., Berkeley, CA,USA) and JMP ver. 8® (SAS Institute, Cary, NC, USA) software, with P < 0.05 considered significant.

## 4. Results

The baseline characteristics of the patients are shown in Table 1. There were no significant differences in age, body mass index (BMI), and duration of hemodialysis (HD) between the diabetic and non-diabetic patients. There were also no significant differences in pre-dialysis serum levels of Ca, P, iPTH, BAP, NTx, and SOS between the two groups. Nor were there differences in medications for chronic kidney disease-mineral and bone disorder (CKD-MBD), hypertension, and anemia between the two groups.

**Table 1:** Baseline Characteristics of Hemodialysis Patients With and Without Type 2 Diabetes a

	Non-Diabetes Group	Diabetes Group	*P* value b
Patients, No.	30	30	
Age b, y	66.6 ± 11.4	65.7 ± 10.6	0.77
50-59, No.	8	9	
60-69, No.	9	10	
70-79, No.	9	8	
80-89, No.	4	3	
BMI b, kg/m^2^	21.2 ± 2.5	21.2 ± 2.0	0.93
Duration of HD b, c, y	5.5 ± 4.9	4.6 ± 5.3	0.47
HbA1c b, %	-	5.73 ± 0.39	
Ca b, mmol/L	2.28 ± 0.17	2.22 ± 0.11	0.12
P b, mmol/L	1.67 ± 0.40	1.70 ± 0.35	0.81
intact PTH b, c, ng/L	100.7 ± 79.6	78.2 ± 52.6	0.2
BAP b, c, μg/L	33.6 ± 19.2	29.9 ± 12.3	0.38
NTx b, c, nmol BCE/L	70.1 ± 27.9	65.4 ± 18.6	0.44
SOS b, c, m/s	1478.4 ± 30.1	1472.4 ± 21.5	0.38
Medication			
CKD c-MBD c (calcium / vitamin D / none)	14/10/6	15/12/3	0.71
Hypertension (ACEI c or ARB c / others / none)	15/5/10	15/2/13	0.62
Anemia (ESA c / none)	25/5	27/3	0.43

^a^Data were analyzed by Mann-Whitney U-test or Chi-squared analysis

^b^Data are expressed as mean ± SD

^c^Abbreviations: ACEI, Angiotensin-converting enzyme inhibitor; ARB, Angiotensin II receptor blocker; ESA, Erythropoiesis stimulating agent; BAP, Bone alkaline phosphatase; HD, Hemodialysis; PTH, Parathyroid hormone; NTx, Telopeptide of type I collagen; SOS, Speed of sound; CKD-MBD, Chronic kidney disease-mineral and bone disorder

**Figure 1:**
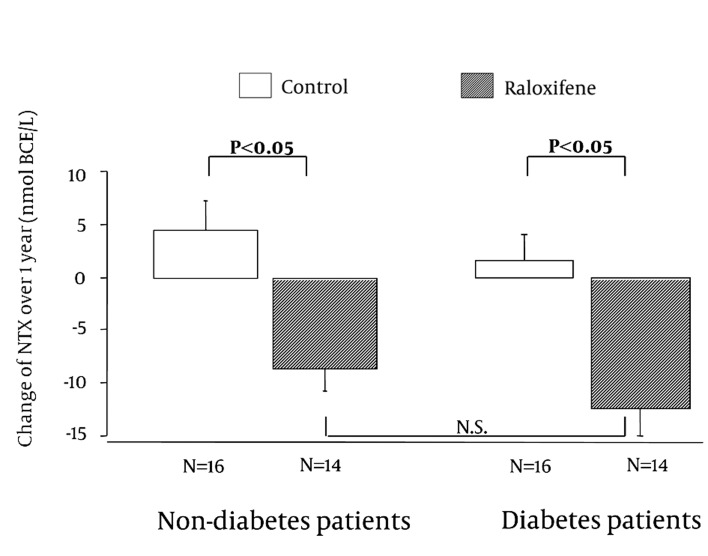
Changes in Serum NTx Concentrations (Value at 1 Year - Pre-Treatment Value) Over 1 Year in Hemodialysis Patients With and Without Diabetes Who Were (Raloxifene Group) and Were Not (Control Group) Treated with Raloxifene Values are shown as means ± SD. Data were analyzed by Mann-Whitney U-test. N.S.: Non Significat

**Figure 2:**
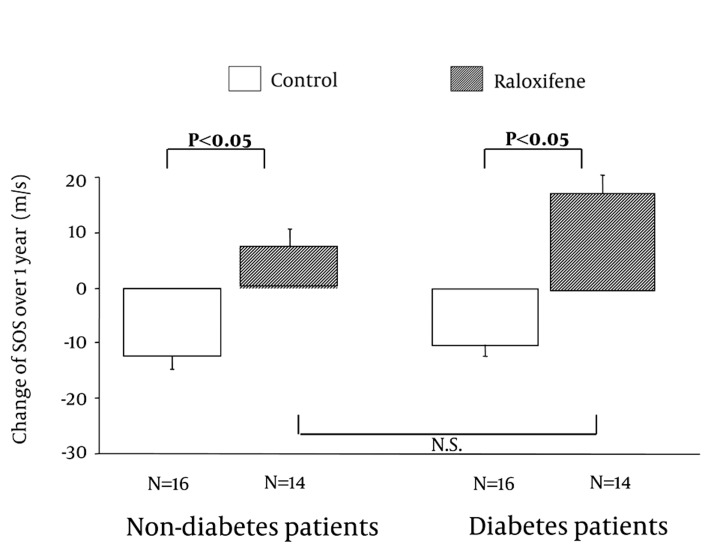
Changes in SOS Values Over 1 Year in Hemodialysis Patients With and Without Diabetes who Were (Raloxifene Group) and Were Not (Control Group) Treated with Raloxifene. Values are shown as means ± SD. Data were analyzed by Mann-Whitney U-test. N.S.: Non Significat

One year after the start of raloxifene treatment, there were no significant differences in bone turnover markers between the control and raloxifene groups among the diabetic hemodialysis patients (Table 2). However, the SOS value in the raloxifene group was significantly higher than that in the control group among the diabetic patients. In the non-diabetic patients, there were no significant differences in bone turnover markers and SOS values between the control and raloxifene groups. Next, we investigated changes in bone turnover markers and SOS values from before to one year after treatment. There was a significant difference in the change in serum NTx (∆NTx) between the raloxifene and control groups in both diabetic and non-diabetic patients (Figure 1). The NTx concentrations increased in both control groups, but decreased in both raloxifene groups after one year of treatment; there was no significant difference in the increment between the diabetic and non-diabetic patients. Changes in SOS (∆SOS) from before to one year after treatment are shown in Figure 2. SOS decreased after one year in both control groups, but increased in both raloxifene groups. The increase in NTx and decrease in SOS in the control groups indicates acceleration of bone resorption with advanced age, suggesting a possible decline in bone density.

**Table 2:** Clinical Parameters (Mean ± SD) Measured After one Year in the Raloxifene and Control Groups in Hemodialysis Patients With and Without Type II Diabetes

	Non-Diabetes Group				Diabetes Group				P value a
	All (n=30)	Control (n=16)	Raloxifene (n=14)	P value a	All (n=30)	Control (n=16)	Raloxifene (n=14)	P value a	
BMI b, kg/m^2^	21.2 ± 2.2	21.3 ± 2.2	21.0 ± 2.4	0.75	21.1 ± 1.8	21.2 ± 2.1	21.1 ± 1.4	0.81	0.99
HbA1c, %	-	-	-	-	5.74 ± 0.73	5.82 ± 0.69	5.64 ± 0.78	0.54	-
Ca b, mmol/L	2.21 ± 0.15	2.21 ± 0.13	2.21 ± 0.17	0.99	2.19 ± 0.15	2.13 ± 0.14	2.27 ± 0.14	0.01	0.59
P b, mmol/L	1.59 ± 0.31	1.58 ± 0.33	1.60 ± 0.30	0.85	1.63 ± 0.29	1.56 ± 0.23	1.71 ± 0.29	0.13	0.59
intact PTH, ng/L	99.2 ± 67.9	104.2 ± 63.8	93.5 ± 74.5	0.67	63.9 ± 40.6	71.9 ± 40.5	54.7 ± 40.1	0.25	0.02
BAP b, μg/L	46.3 ± 29.8	49.4 ± 27.5	42.8 ± 32.9	0.55	49.9 ± 21.5	51.8 ± 25.2	47.8 ± 16.9	0.61	0.58
NTx b, nmol BCE/L	68.9 ± 28.4	74.2 ± 33.7	62.7 ± 20.2	0.27	60.6 ± 22.4	63.5 ± 25.5	57.2 ± 18.7	0.44	0.21
SOS b, m/s	1475.8 ± 34.6	1465.5 ± 34.9	1487.6 ± 31.3	0.08	1474.1 ± 26.2	1464.2 ±12.0	1485.4 ± 33.4	0.02	0.82

^a^Data between the control and raloxifene group and between the non-diabetes and diabetes groups were analyzed by Mann-Whitney U-test.

^b^Abbreviations: BMI, Body mass index; Ca, Calcium; P, Phosphorus; PTH, Parathyroid hormone; NTx, N-terminal cross-linking telopeptide of type I; SOS, Speed of sound; BAP, Bone alkaline phosphatase

These results suggest that raloxifene suppresses bone resorption and increases SOS in both non-diabetic and diabetic female hemodialysis patients. The response to raloxifene did not differ significantly between diabetic and non-diabetic patients. During follow-up of the patients who received raloxifene, no cases of breast cancer, cervical carcinoma, or cerebral or myocardial infarction caused by thrombosis were detected.

## 5. Discussion

The disordered glucose metabolism that occurs in diabetes has a major effect on bone metabolism, even in hemodialysis patients with renal osteodystrophy. Hemodialysis patients with diabetes are more likely to have adynamic bone disease (diagnosed as low bone turnover) than non-diabetic hemodialysis patients (1). This low bone turnover in diabetes may be due to direct suppression of PTH secretion, since it has been shown in vitro that a high glucose concentration impairs the function of parathyroid cells and reduces secretion of PTH (14). Low bone turnover is also thought to be promoted by reduced PTH secretion caused by nutritional deficiency in diabetic patients. In our study, there were no significant differences in the values of NTx (a bone resorption marker), BAP (a bone formation marker), iPTH, and SOS between diabetic and non-diabetic patients at baseline, but iPTH in diabetic patients was lower than in non-diabetic patients after 1 year, which has also been reported previously. Kikunami et al. reported that the decrement in PTH levels in diabetic hemodialysis patients depended on the duration of hemodilaysis (15). Clinically, Inaba et al. showed that the serum PTH level in diabetic patients undergoing hemodialysis is significantly lower than that in non-diabetic hemodialysis patients (16). Another study showed that osteoblast function is impaired by apoptosis induced by advanced glycation end-products (17).

Administration of estrogen has been shown to significantly increase BMD in lumbar vertebrae after one year in postmenopausal dialysis patients (18). Estrogen has a well-known osteoprotective action and suppresses activation of osteoclasts by PTH (19). In contrast, the action of PTH on bone resorption is enhanced under the condition of estrogen deficiency (20). In general, hemodialysis patients have secondary hyperparathyroidism. In female hemodialysis patients, bone resorption might be accelerated by menopause (estrogen deficiency), and there is a high risk of further decline in BMD compared to male hemodialysis patients. Taken together, these findings suggest that administration of estrogen might be effective for inhibition of bone resorption in female hemodialysis patients. Raloxifene is an estrogen agonist in bone that is currently approved for the prevention and treatment of osteoporosis. SERMs are thought to increase bone mass volume by binding to the estrogen receptor (ERα) on osteoclasts and inhibiting production of Fas ligand in these cells (21). Binding of the Fas ligand to the Fas receptor induces apoptosis of osteoclasts, and therefore binding of a SERM to the ERα receptor suppresses apoptosis and bone absorption.

Studies specifically targeting osteoporosis in individuals with diabetes have not been performed, and therefore treatment recommendations for osteoporosis in diabetic patients follow other guidelines, such as those for postmenopausal women in the general population. Moreover, there are no guidelines for the treatment of osteoporosis in hemodialysis patients with diabetes. To the best of our knowledge, studies specifically targeting osteoporosis in diabetic hemodialysis patients have not been performed, and our study is the first interventional study targeting osteoporosis in diabetic hemodialysis patients.

Our study was limited by the small number of subjects, but we did find that treatment with raloxifene for one year increased SOS in both diabetic and non-diabetic hemodialysis patients. This result suggests that raloxifene is beneficial for all menopausal hemodialysis patients, regardless of the presence of diabetes. However, BMD itself does not predict fracture and mortality in dialysis patients, in contrast to findings in the general population (12). The KDIGO clinical practice guidelines for CKD-MBD recommend that secondary hyperparathyroidism should be addressed first in bone disease in dialysis patients (22). An additional examination by bone biopsy is recommended prior to therapy with antiresorptive agents in dialysis patients with low BMD, but this is a difficult and invasive procedure. Both renal osteodystrophy and osteoporosis can lead to increased bone fragility and fracture. Therefore, suppression of the decrease in BMD by raloxifene might be beneficial for decreasing bone fragility in hemodialysis patients with low BMD.

This study had particular limitations. First, it was not population based, nor were all participants enrolled from identical populations; thus, selection bias could have occurred. The sample was also not large enough to make any definitive conclusions. Second, the patients enrolled in this study were treated at Oyama Suginoki Clinic only, and might not be representative of average Japanese diabetic hemodialysis patients.

In conclusion, this study suggests that the reduction in BMD caused by postmenopausal osteoporosis is involved in the development of bone disease in female hemodialysis patients with type 2 diabetes, and that raloxifene hydrochloride can improve low BMD in these patients as well as in non-diabetic female hemodialysis patients.

## References

[1] Sherrard DJ, Hercz G, Pei Y, Maloney NA, Greenwood C, Manuel A (1993). The spectrum of bone disease in end-stage renal failure--an evolving disorder.. Kidney Int..

[2] Bouillon R (1991). Diabetic bone disease.. Calcif Tissue Int..

[3] Olmos JM, Perez-Castrillon JL, Garcia MT, Garrido JC, Amado JA, Gonzalez-Macias J (1994). Bone densitometry and biochemical bone remodeling markers in type 1 diabetes mellitus.. Bone Miner..

[4] Adami S (2009). Bone health in diabetes: considerations for clinical management.. Curr Med Res Opin..

[5] Sharifi F, Ahmadimoghadam N, Mousavinasab N, Amani R, Mostafavi A, Agin K (2006). The Relationship Between Type 2 Diabetes Mellitus And Bone Density In Postmenopausal Women.. Int J Endocrinol Metab..

[6] Weisinger JR, Gonzalez L, Alvarez H, Hernandez E, Carlini RG, Capriles F (2000). Role of persistent amenorrhea in bone mineral metabolism of young hemodialyzed women.. Kidney Int..

[7] Clarkson TB (2002). Raloxifene revisited.. Fertil Steril..

[8] Hernandez E, Valera R, Alonzo E, Bajares-Lilue M, Carlini R, Capriles F (2003). Effects of raloxifene on bone metabolism and serum lipids in postmenopausal women on chronic hemodialysis.. Kidney Int..

[9] Ishani A, Blackwell T, Jamal SA, Cummings SR, Ensrud KE (2008). The effect of raloxifene treatment in postmenopausal women with CKD.. J Am Soc Nephrol..

[10] Saito O, Saito T, Asakura S, Sugase T, Ito C, Ando Y (2011). The effects of raloxifene on bone turnover markers and bone mineral density in women on maintenance hemodialysis.. Clin Exp Nephrol..

[11] Maeno Y, Inaba M, Okuno S, Yamakawa T, Ishimura E, Nishizawa Y (2005). Serum concentrations of cross-linked N-telopeptides of type I collagen: new marker for bone resorption in hemodialysis patients.. Clin Chem..

[12] Kishimoto H (2004). [Cm-100].. Nihon Rinsho..

[13] Jamal SA, Chase C, Goh YI, Richardson R, Hawker GA (2002). Bone density and heel ultrasound testing do not identify patients with dialysis-dependent renal failure who have had fractures.. Am J Kidney Dis..

[14] Sugimoto T, Ritter C, Morrissey J, Hayes C, Slatopolsky E (1990). Effects of high concentrations of glucose on PTH secretion in parathyroid cells.. Kidney Int..

[15] Kikunami K, Nishizawa Y, Tabata T, Nakatsuka K, Matsushita Y, Inoue T (1990). Changes in parathyroid hormone in diabetic patients on long-term hemodialysis.. Nephron..

[16] Inaba M, Nagasue K, Okuno S, Ueda M, Kumeda Y, Imanishi Y (2002). Impaired secretion of parathyroid hormone, but not refractoriness of osteoblast, is a major mechanism of low bone turnover in hemodialyzed patients with diabetes mellitus.. Am J Kidney Dis..

[17] Ogawa N, Yamaguchi T, Yano S, Yamauchi M, Yamamoto M, Sugimoto T (2007). The combination of high glucose and advanced glycation end-products (AGEs) inhibits the mineralization of osteoblastic MC3T3-E1 cells through glucose-induced increase in the receptor for AGEs.. Horm Metab Res..

[18] Matuszkiewicz-Rowinska J, Skorzewska K, Radowicki S, Sokalski A, Przedlacki J, Niemczyk S (1999). The benefits of hormone replacement therapy in pre-menopausal women with oestrogen deficiency on haemodialysis.. Nephrol Dial Transplant..

[19] Riggs BL (2000). The mechanisms of estrogen regulation of bone resorption.. J Clin Invest..

[20] Grey AB, Stapleton JP, Evans MC, Reid IR (1996). Accelerated bone loss in post-menopausal women with mild primary hyperparathyroidism.. Clin Endocrinol (Oxf)..

[21] Nakamura T, Imai Y, Matsumoto T, Sato S, Takeuchi K, Igarashi K (2007). Estrogen prevents bone loss via estrogen receptor alpha and induction of Fas ligand in osteoclasts.. Cell..

[22] (2009). KDIGO clinical practice guideline for the diagnosis, evaluation, prevention, and treatment of Chronic Kidney Disease-Mineral and Bone Disorder (CKD-MBD).. Kidney Int Suppl..

